# Financial burden among cancer patients: A national‐level perspective

**DOI:** 10.1002/cam4.5049

**Published:** 2022-07-19

**Authors:** Dinesh Pal Mudaranthakam, Jo Wick, Elizabeth Calhoun, Tami Gurley

**Affiliations:** ^1^ Department of Biostatistics & Data Science University of Kansas Medical Center Kansas City Kansas USA; ^2^ The University of Kansas Cancer Center Kansas City Kansas USA; ^3^ Department of Population Health, Health Policy & Management University of Kansas Medical Center Kansas City Kansas USA

**Keywords:** cancer treatment, financial burden, financial challenges, treatment burden

## Abstract

**Background:**

This research study aimed to evaluate the financial burden among older cancer patients and its corresponding risk factors. Factors such as increasing treatment costs and work limitations often lead cancer patients to bankruptcy and poor quality of life. These consequences, in turn, can cause higher mortality rates among these patients.

**Methods:**

This retrospective cohort study utilized data from the Health Retirement Study (HRS), conducted by the University of Michigan (*N* = 18,109). Eligible participants had responses captured from years 2002 to 2016. Participants were classified according to any self‐reported cancer diagnosis (yes or no) and were compared on the basis of financial, work, and health‐related outcomes. Propensity score (PS) matching was applied to reduce the effects of potential confounding factors. Also only, individuals with an age ≥50 and ≤85 during Wave 6 were retained.

**Results:**

Multivariate analysis with random effects revealed several indicators of financial burden when comparing participants with a cancer diagnosis to those with no history of cancer. Mean out‐of‐pocket costs associated with a cancer diagnosis were $1058 higher when compared to participants with no history of cancer, suggesting that even cancer patients with insurance coverage faced out‐of‐pocket costs. Respondents with cancer patients had higher odds of encountering financial hardship if they are facing Work Limitations (OR = 2.714), Regular use of Medications (OR = 2.518), Hospital Stays (OR = 2.858), Declining Health (OR = 2.349), or were being covered under government health insurance (OR = 5.803) than respondents who did not have cancer, or suffered from mental health issues such as Depression (OR = 0.901).

**Conclusion:**

Cancer patients contend with increasing financial costs during their treatment. However, most newly diagnosed patients are not aware of these costs and are given few resources to handle them.

## Graphical abstract

Given that this study cohort is primarily senior citizens, it is especially troubling that an active working status seems to be an important requirement for managing cancer treatment without severe financial hardship. In the absence of more robust financial support for these patients, financial planning has become a vital part of the treatment process for cancer patients. Until the introduction of legislation that makes cancer treatment financially viable, health systems have a responsibility to direct resources toward the education and assistance of these patients.

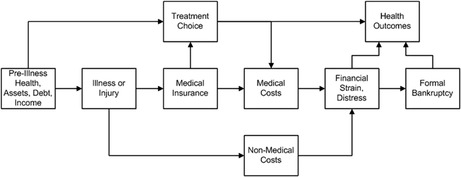

## 1 INTRODUCTION

Cancer is a leading cause of death and disability in the world.^1^ In the United States, 87% of cancer diagnoses occurring in patients 50 years or older.^2^ Regular screening detects cancer earlier and provides patients with more treatment options and better chances of remission. In combination with early detection, modern cancer treatments have made cancer survival increasingly feasible. Successful treatment, however, is more difficult to achieve depending on comorbidity and cancer stage.^3^

In recent years, cancer patients have faced a significant challenge in handling the cost of treatment. A lack of regulation regarding prescription drug price increases, value‐based pricing, and patent abuse has led to skyrocketing treatment costs in the United States.^4^ These costs typically present in the form of higher out‐of‐pocket costs, medication costs, and care costs.^5^ Before 2000, the average price of cancer drugs for a year of treatment was between $5000 and $10,000. By 2012, it increased to more than $100,000.^6^ These costs can be even more severe for older patients who have a limited source of income.

Additionally, cancer drugs are the most expensive medications among those commonly prescribed to Medicare part D patients.^7^ Gaps in prescription drug coverage have further complicated matters for cancer patients and significantly increased out‐of‐pocket costs. As treatment becomes prohibitively expensive, patients have resorted to declining or becoming uncompliant with treatment.^8^ Cost‐related noncompliance in cancer patients has been tied to several factors, including race, source of insurance, and sex. Marginalized communities, such as older African American men, Medicare beneficiaries, and uninsured patients, are particularly affected.^9^ However, cost‐related noncompliance is not limited to these patients. It is estimated that greater than 20% of all cancer survivors have delayed or missed care in the past year due to cost.^10^

Financial toxicity is a term first coined in 2009 to describe the clinical relevance of financial distress, particularly in cancer treatment.^11^ Financial toxicity, as a topic of research, emphasizes that severe financial distress can limit a patient's ability to continue treatment and lead to severe consequences for patient outcomes. The severity of financial toxicity among cancer patients has gained some attention of late, including the development of educational materials by the National Cancer Institute.^9^ Through this study, The University of Kansas Cancer Center began developing a foundation specifically for investigating factors contributing to the financial burden of cancer patients. While there is a wealth of research on financial burden among older populations, as well as financial burdens among cancer patients, the intersection of these two factors has been less investigated. To investigate this intersection, we required a dataset that had a sufficient sample of cancer participants as well as participants without cancer for comparison. In consideration of this, we decided to investigate the financial burden of cancer diagnosis and treatment specifically among older cancer patients when compared to older populations without cancer. Our secondary aim was to identify factors such as treatment costs and prescription costs which could contribute to the increased medical costs of cancer patients compared to non‐cancer patients. Identifying these factors could form the basis for further investigation on how these factors contribute to financial toxicity.

We have used the data made available through the Health and Retirement Study to conduct our initial research. We have used the data made available through the Health and Retirement Study to conduct our initial research.

## 2 METHODS

**FIGURE 1:**
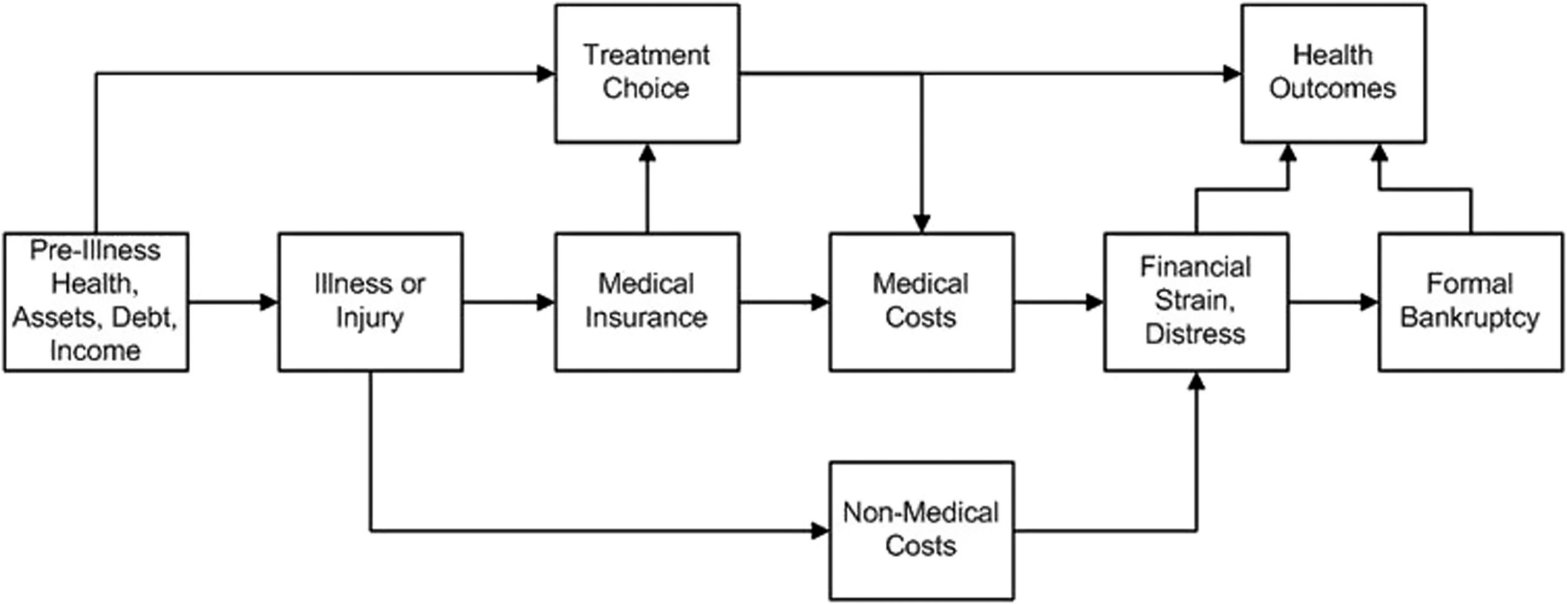
Conceptual framework; credit: Scott Ramsey, MD, PhD

Financial toxicity factors were chosen based on the existing literature and National Cancer Institute guidelines. Dr. Scott Ramsey designed a conceptual framework to illustrate the complex inter‐related nature of the various factors that could lead a cancer patient toward financial hardship or financial burden.^12^ The conceptual framework is described in Figure 1.^13^, ^14^

To study financial toxicity among cancer patients, we used the University of Michigan Health and Retirement Study (HRS) (RAND Version). The RAND HRS longitudinal data file is a clean and easy‐to‐use version that includes 13 waves of core interview data across 15 survey years and exit interview data.^15^ The data were derived from a longitudinal cohort panel study that surveyed participants 50 years or older between 2002 and 2016.^15^ Approximately 20,000 participants across America participated and are a representative sample of the population.^16^ The baseline interview was conducted in‐person and the follow up survey data through telephone. Follow‐up was conducted biannually. Since the data set is de‐identified, it was exempted from human subject's protections.

### 2.1 Statistical analysis

For our study to retain consistent measures, our sample included the responses captured from 2002 to 2016. Seventy five percent of the participants had at least 10 years' worth of data spanning six waves. Among these participants, the data set included variables related to cancer diagnosis, demographics, socioeconomic status, work history, utilization of health services, and finances. The participants baseline demographic characteristics are depicted in Table 1. Participants were stratified based on personal history of cancer (yes/no) and only participants aged 50–85 at the Wave 6 response were retained.

**TABLE 1:** Participantd demographics at baseline

Variable	History of cancer	No history of cancer	Total	Chi‐square	Fisher's exact	*t*‐test
Total participants	2444 (13.5%)	15,665 (86.5%)	18,109 (100%)			
Gender				0.056	0.054	
Male	1047 (42.84%)	6387 (40.77%)	7434 (41%)			
Female	1397 (57.16%)	9278 (59.23%)	10,675 (59%)			
Race				<0.001	<0.001	
White/caucasian	2122 (86.82%)	12,796 (81.71%)	14,918 (82%)			
Black/african american	256 (10.47%)	2247 (14.35%)	2503 (14%)			
Other	66 (2.7%)	617 (3.94%)	683 (4%)			
Hispanic				<0.001	<0.001	
Not hispanic	2324 (95.09%)	14,321 (91.44%)	16,645 (92%)			
Hispanic	120 (4.91%)	1341 (8.56%)	1461 (8%)			
Ever smoked				<0.001	<0.001	
No	911 (37.6%)	6570 (42.37%)	7481 (41%)			
Yes	1512 (62.4%)	8937 (57.63%)	10,449 (58%)			
Census region				0.234	0.202	
Northeast	411 (16.82%)	2548 (16.29%)	2959 (16%)			
Midwest	623 (25.5%)	3896 (24.9%)	4519 (25%)			
South	952 (38.97%)	6473 (41.37%)	7425 (41%)			
West	455 (18.62%)	2717 (17.37%)	3172 (18%)			
Other	2 (0.08%)	12 (0.08%)	14 (0%)			
Current marital status: with partnership				NaN	<0.001	
Married	1442 (59%)	9769 (62.36%)	11,211 (62%)			
Married, spouse absent	23 (0.94%)	143 (0.91%)	166 (1%)			
Separated	29 (1.19%)	261 (1.67%)	290 (2%)			
Divorced	225 (9.21%)	1542 (9.84%)	1767 (10%)			
Separated/divorced	0 (0%)	0 (0%)	0 (0%)			
Widowed	642 (26.27%)	3499 (22.34%)	4141 (23%)			
Never married	83 (3.4%)	451 (2.88%)	534 (3%)			
Unknown	0 (0%)	0 (0%)	0 (0%)			
Education: degrees, diplomas				0.246	0.174	
No degree	289 (19.2%)	2295 (22.39%)	2584 (14%)			
GED	74 (4.92%)	482 (4.7%)	556 (3%)			
HS	497 (33.02%)	3212 (31.34%)	3709 (20%)			
HS/GED	257 (17.08%)	1732 (16.9%)	1989 (11%)			
AA/LT BA	66 (4.39%)	385 (3.76%)	451 (2%)			
BA	180 (11.96%)	1256 (12.26%)	1436 (8%)			
MA/MBA	102 (6.78%)	631 (6.16%)	733 (4%)			
Law/MD/PhD	39 (2.59%)	252 (2.46%)	291 (2%)			
Other	1 (0.07%)	3 (0.03%)	4 (0%)			
Education: categorical summary				0.096	0.086	
<High‐school	289 (19.22%)	2295 (22.4%)	2584 (14%)			
GED	74 (4.92%)	482 (4.7%)	556 (3%)			
High‐school graduate	497 (33.05%)	3228 (31.51%)	3725 (21%)			
Some college	323 (21.48%)	2101 (20.51%)	2424 (13%)			
College and above	321 (21.34%)	2139 (20.88%)	2460 (14%)			
Unemployed				0.138	0.119	
No	581 (97.32%)	5464 (95.99%)	6045 (33%)			
Yes	16 (2.68%)	228 (4.01%)	244 (1%)			
Covered by health insurance from a current or previous employer				0.003	0.003	
No	1645 (68.29%)	10,096 (65.16%)	11,741 (65%)			
Yes	764 (31.71%)	5398 (34.84%)	6162 (34%)			
Covered by federal government health insurance program				<0.001	<0.001	
No	517 (21.25%)	5670 (36.28%)	6187 (34%)			
Yes	1916 (78.75%)	9959 (63.72%)	11,875 (66%)			
Covered by other health insurance				<0.001	<0.001	
No	1824 (75.72%)	12,533 (80.87%)	14,357 (79%)			
Yes	585 (24.28%)	2964 (19.13%)	3549 (20%)			
Whether retired: consider self‐retired				<0.001	<0.001	
Not retired	368 (17.22%)	3966 (28.95%)	4334 (24%)			
Completely retired	1202 (56.25%)	5823 (42.5%)	7025 (39%)			
Partly retired	196 (9.17%)	1503 (10.97%)	1699 (9%)			
Question irrelevant	371 (17.36%)	2409 (17.58%)	2780 (15%)			
Education: years of education	12.689	12.357	12.394			<0.001
Total wealth (including secondary residence)	436,097.775	377,377.052	385,097.008			0.019
Total non‐housing wealth	300,606.893	257,392.148	263,156.218			0.057
Total household income (respondent & spouse)	51,212.250	53,214.171	52,883.513			0.226
Individual income from social security DI or SSI	320.410	405.959	395.068			0.023
Individual income from social security retirement	7506.370	5804.919	6035.354			<0.001
Whether retired: retirement month, if says retired	6.058	5.978	5.991			0.461
Age	71.699	67.841	68.367			<0.001

Study outcomes include measures of the financial burden and impact of cancer and cancer treatment. The variables “change in assets” and “change in debt” represent financial changes that have occurred since a participant's last interview. A value of 0 indicates that a patient has no outstanding debt. The “out of pocket costs” variable represents the expenses incurred for medical care and was recorded as a continuous numerical variable. Other continuous financial variables were total wealth, total household income, individual income from Social Security Disability Insurance (SSDI) or Supplemental Security Income (SSI), and individual income from social security retirement. Categorical variables included work limitation due to diagnosis, hospital stay, depression, regular use of medication, declining health, work status, coverage by other insurance plans, coverage by federal health insurance program, and coverage by health insurance from a current or previous employer.

Propensity score (PS) matching was used to reduce the effects of potential confounding variables among cancer and non‐cancer groups. PS matching reduces bias due to confounding factors by matching patient characteristics on baseline variables using a multivariable logistic regression model. The greedy algorithm matched every respondent with a cancer diagnosis with three respondents with no cancer diagnosis using a caliper of 0.25. The matching algorithm included race, ethnicity, smoking status, household income, age, and marital status variables. Post matching, the total sample included 8772 participants, of which 2193 were respondents with a cancer diagnosis. Post PS, cancer and non‐cancer groups were balanced with regards to covariates. GLS random effects regression model was used to evaluate differences in continuous measures of financial burden and between patients with and without a diagnosis of cancer. Logistic regression models were used to evaluate the correlation between categorical measures of financial burden and patient history of cancer diagnosis. All analysis was performed using SAS 9.4 M7 and STATA v16.

## 3 RESULTS

Differences in continuous measures of financial burden are shown in Table 2. Participants with no diagnosis of cancer reported a greater increase in assets when compared to those with cancer. The average increase in assets among respondents with a cancer diagnosis was $3758 lower than respondents with no cancer history, although these results were not statistically significant (95% CI $19,272–$11,756; *p* = 0.457). Respondents with a cancer diagnosis owed $138.52 less to financial institutions than respondents with no cancer diagnosis, but these results were also not statistically significant (95% CI $307–$548; *p* = 0.183). Additionally, participants with a history of cancer spent more money on their health care. The average out‐of‐pocket cost was $1058 more among the respondents with a cancer diagnosis than the participants with no cancer history (95% CI $872–$1243, *p* < 0.001).

**TABLE 2:** Univariate linear regression analysis

Financial burden (Y)	Participants with diagnosis of cancer (*n* = 2387) (mean)	Participants with no diagnosis of cancer (*n* = 11,689) (mean)	Mean difference	*p*‐value	95% confidence interval	
Change in total wealth	$562,474.7	$525,217.8	$37,256.9	0.834	−$24,315.94	$30,135.66
Change in debt	$3117.82	$2502.38	$615.44	0.111	−$84.06	$811.99
Total household income	$57,663.68	$53,907.41	$3756.27	0.553	−$1445.29	$2698.03
Individual income from social security DI or SSI	$274.17	$275.26	−$1.09	0.461	−$52.28	$23.78
Individual income from social security retirement	$10,402.56	$9803.96	$598.6*	<0.001	$614.96	$903.35
Out of pocket cost	$4809.99	$4130.08	$679.91*	0.003	$149.79	$758.22

* *p* < 0.001.

Logistic regression analysis identified several contributing factors that were associated with a cancer diagnosis in older respondents. As shown in Table 3, these factors included work limitations (OR = 2.714, 95% CI = 2.235–3.296, *p* < 0.001), hospital stays (OR = 2.858, 95% CI = 2.417–3.380, *p* < 0.001), regular use of medications (OR = 2.518, 95% CI = 1.858–3.412, *p* < 0.001), declining health (OR = 2.349, 95% CI = 1.962–2.813, *p* < 0.001), covered by federal government health insurance program (OR = 5.830, 95% CI = 4.428–7.675, *p* < 0.001), covered by health insurance from current or previous employee (OR = 0.485, 95% CI = 0.382–0.614, *p* < 0.001).

**TABLE 3:** Multivariate analysis with random effects logistic regression analysis

Financial burden (year)	Participants with diagnosis of cancer (mean)	Participants with no diagnosis of cancer (mean)	Odd ratio	95% confidence interval	
Work limitation	43.45%	34.55%	2.714**	2.235	3.296
Hospital stay	40.47%	28.65%	2.858**	2.417	3.380
Depression	14.25%	13.67%	0.901	0.705	1.151
Regular use of medications	91.60%	85.64%	2.518**	1.858	3.412
Declining health	36.78%	28.13%	2.349**	1.962	2.813
Whether retired	69.61%	58.90%	0.828*	0.739	0.928
Covered by other health Insurance	22.63%	21.02%	0.700*	0.553	0.886
Covered by federal government health insurance program	88.04%	78.29%	5.830**	4.428	7.675
Covered by health insurance from current or previous employee	23.92%	26.67%	0.485**	0.382	0.614

** *p* < 0.001.

* *p* ≤ 0.05.

## 4 DISCUSSION

The primary aim of this analysis was identifying the financial burden of individuals with cancer compared to individuals who do not have cancer. Additionally, the secondary aim was to analyze the relative presence of established risk factors in financial toxicity between these two groups. Through these two aims we could investigate how established trends in financial toxicity among cancer patients of all ages related to older cancer patients specifically.

Older cancer patients had higher odds of hospital stays, declining health and regular use of medication when compared to older patients without cancer. This is consistent with research investigating adverse outcomes relating to physical health among cancer patients of all ages.^17^ Previous research has demonstrated that family and group support might help these cancer patients overcome treatment‐related depression.^18^ However, even with propensity score matching that included marital status there was a significant difference in the odds of depression between the two groups. This does not take into consideration the presence or absence of additional family support beyond a spouse.

In the United States, 66.5% of individuals who filed for bankruptcy cited illness or medical expenses as contributing factors.^19^ These considerations are amplified for cancer patients, among whom 42% will fully deplete their assets by the second year of their diagnosis.^20^ The results of our analysis indicated that out of pocket healthcare costs were significantly higher for older cancer patients, and that older cancer patients had higher odds of utilizing expensive healthcare avenues such as hospitalization and medications compared to older patients without cancer. Given that the cost of these aspects of treatment could be even more straining on individuals with a fixed income, it would be valuable to evaluate bankruptcy claims and financial depletion in conjunction with these results among the older populations. These treatment aspects and their cost should also be a consideration in non‐adherence discussions for older patients with cancer. Additional consideration can be given to the significantly higher odds of experiencing work limitations for older respondents with cancer when compared to those without cancer. When the personal cost of treatment adherence can have a significantly detrimental effect on the financial stability of a cancer patient and their family, non‐adherence may be seen as a preferable alternative.

Regarding healthcare policy, it is apparent that even minor changes emphasizing transparency and open communication could make a crucial difference in minimizing the financial burden among cancer patients. For example, the excessive use of CT scans has been shown to have minimal impact on the survival rate for patients with large B‐cell lymphoma.^21^ Also, pre‐operative chemotherapy is being prescribed to breast cancer patients who do not meet the suggested treatment categories.^22^ Previous research has established that financial burden can contribute worse outcomes among cancer patients of all ages. Further analysis will be crucial in determining how financial burden affects health outcomes among cancer patients, and what treatment decisions can mitigate that harm.

From a patient perspective, there are several issues most associated with financial toxicity.^23^, ^24^ These issues include a lack of open communication during treatment planning, the cost of treatment, vague disclosure of financial assistance options, unclear insurance reimbursement procedures, and gaps in insurance coverage. A study showed that only 28% of clinicians were comfortable discussing out‐of‐pocket costs with their patients.^25^ Also, it appears that many patients are not being made aware of the possible financial assistance that their healthcare systems and pharmaceutical producers can provide.^26^ With a lack of clarity on how insurance reimbursements work, many of the latest therapies are prescribed despite a lack of insurance coverage (genomics, yoga). In many states, insurance companies can deny payment for therapeutic clinical trials.^27^ Our analysis indicated higher odds of government health insurance coverage, and lower odds of health insurance coverage from a current or previous employer among older respondents with cancer compared to older respondents without cancer. Navigating these treatment decisions with an additional consideration of the patient's health insurance coverage would be a valuable consideration.

Patients and their care teams must have a transparent and open discussion regarding the financial aspects of treatment. Every health care system should have a team of financial professionals familiar with health insurance policies, the Affordable Care Act, Medicaid, and Medicare. Every cancer patient should go through this pre‐treatment discussion with the financial professional and discuss clearly and openly what is going on with their lives and how the health system could help them either with a payment plan, a 2nd insurance plan, or even potential adjustments to their insurance policy. Even for Medicare patients, it is not straightforward as there is wide variability in coverage.^28^ The financial and health care teams can map out the treatment plan and work with the patient to understand the expected insurance coverage and expected out‐of‐pocket costs to help eliminate devasting financial consequences. This approach is no different from the financial professionals who help individuals with their retirement planning. The return on the investment for the health system is substantial, given that patients can more reliably pay for their treatment and other costs such as out‐of‐pocket expenses, deductibles, and copays. Health care systems should take a proactive rather than a reactive approach and work out a plan with the patient and the care team. As patients progress with their treatment, the patient or the caregiver should be updated at each step as treatment changes.

Patients and their families also have equal responsibility to avoid financial burdens. Patients must be open with their communication and let their health care team know what is going on in their life. This would include situations where patients are unable to afford insurance premiums or change their insurance plans. These open communications can minimize the back and forth and ease the administrative and claims process.^29^ This, in turn, allows the health care team to explore other options by working with their financial teams. With the financial uncertainty that occurs post‐cancer diagnosis, patients should rely on their care team to help navigate the difficult terrain ahead.

Cancer centers and health care systems are more frequently utilizing financial counselors, financial navigators, patient navigators, and social workers to help patients navigate their cancer treatment.^30^, ^31^ Financial counselors and navigators can help determine current benefits, explain insurance benefits, help look for additional eligible benefits, and help with setting up payment plans. On the other hand, patient navigators and social workers can assist patients in finding resources to navigate the cancer journey, such as transportation, childcare, utility assistance, and prescription assistance.^30^ Additionally, connecting patients with resources such as cancer support groups can have beneficial effects on mental health outcomes during the treatment process.^32^

Cancer patients should not be forced to sacrifice their financial stability for the sake of their treatment. Due to the dynamic nature of financial toxicity, detailed intervention‐based research must be conducted to determine the best strategies to avoid substantial financial burdens. Further research should investigate whether financial toxicity occurs more often in certain cancer types, geographies, insurance types, provider specialties, and other factors influencing costs.

### 4.1 Limitation

Our data set lacked fields such as complete earnings and other financial savings information. Only cumulative information around change in assets and debt were included. Additionally, the data set did not include detailed information about cancer type, treatment, and outcomes. We cannot know if the person retired or gained access to Medicare because of their cancer and whether all the out‐of‐pocket costs are directly attributable to their cancer. However, there were significantly more out‐of‐pocket costs for cancer patients compared to that of non‐cancer patients. Even with insurance coverage, cancer patients had higher odds of encountering financial hardship. Lack of information for other comorbidities restricts us to assume that overall financial changes are related to cancer diagnosis as these patients could also be suffering from other medical bills.

## AUTHOR CONTRIBUTIONS

Dinesh Pal Mudaranthakam oversaw all aspects of drafting, revision, and final approval of the manuscript. Jo Wick and Tami Gurley guided Dinesh Pal Mudaranthakam around the Statistical and Analysis Plan (SAP). Elizabeth Calhoun oversaw the policy and patient safety aspect that arouse from the analysis.

## FUNDING INFORMATION

The author(s) received no financial support for the research, authorship, and/or publication of this article.

## CONFLICT OF INTEREST

The author(s) declared no potential conflicts of interest with respect to the research, authorship, and/or publication of this article.

## ETHICS APPROVAL

The University of Kansas Medical Center granted approval under a central IRB with reliance by the other institutions (STUDY00147028).

## Data Availability

Publicly available data set through the Health and Retirement Study portal was utilized for this research. https://hrs.isr.umich.edu/about.

## References

[1] Wild C , Stewart B . World Cancer Report 2014. World Health Organization; 2014:482‐494.

[2] American Society of Cancer . Cancer Facts and Figures. American Cancer Society; 2017.

[3] Wender RC , Brawley OW , Fedewa SA , Gansler T , Smith RA . A blueprint for cancer screening and early detection: advancing screening's contribution to cancer control. CA Cancer J Clin. 2019;69(1):50‐79. 30452086 10.3322/caac.21550

[4] Vincent RS . The high cost of prescription drugs: causes and solutions. Blood Cancer J. 2020;10(6):71. 32576816 10.1038/s41408-020-0338-x PMC7311400

[5] Lyman GH , Kuderer N . Financial toxicity, financial abuse, or financial torture: let's call it what it is. 2020. 10.1080/07357907.2020.1735084 32093505

[6] Kantarjian H , Rajkumar SV . Why are cancer drugs so expensive in the United States, and what are the solutions? Mayo Clin Proc. 2015;90(4):500‐504. doi:10.1016/j.mayocp.2015.01.014 25792242

[7] Cubanski J , Rae M , Young K , Damico A . How Does Prescription Drug Spending and Use Compare Across Large Employer Plans, Medicare Part D, and Medicaid. Kaiser Family Foundation; 2019.

[8] Lee MJ , Khan MM , Salloum RG . Recent trends in cost‐related medication nonadherence among cancer survivors in the United States. J Manag Care Spec Pharm. 2018;24:56‐64. 29290172 10.18553/jmcp.2018.24.1.56 PMC10398090

[9] Chhatwal J , Mathisen M , Kantarjian H . Are high drug prices for hematologic malignancies justified? A critical analysis. Cancer. 2015;121(19):3372‐3379. 26102457 10.1002/cncr.29512

[10] Sabatino SA , Coates RJ , Uhler RJ , Alley LG , Pollack LA . Health insurance coverage and cost barriers to needed medical care among U.S. adult cancer survivors age<65 years. Cancer. 2006;106:2466‐2475. 16639732 10.1002/cncr.21879

[11] O'Connor JM , Kircher SM , de Souza JA . Financial toxicity in cancer care. J Community Support Oncol. 2016;14(3):101‐106. doi:10.12788/jcso.0239 27058866

[12] PDQ Adult Treatment Editorial Board . Financial Toxicity and Cancer Treatment (PDQ®): Health Professional Version. PDQ Cancer Information Summaries. 2021.

[13] National Cancer Institute . Financial Toxicity and Cancer Treatment (PDQ®)–Health Professional Version. 2017. 27583328

[14] Hazra A . Using the confidence interval confidently. J Thorac Dis. 2017;9(10):4125‐4130. 10.21037/jtd.2017.09.14 29268424 PMC5723800

[15] Fisher GG , Ryan LH . Overview of the health and retirement study and introduction to the special issue. Work Aging Retire. 2018;4:1‐9. 29423243 10.1093/workar/wax032 PMC5798643

[16] Smith R , Clarke L , Berry K , et al. A comparison of methods for linking health insurance claims with clinical records from a large cancer registry. Med Decis Making. 2001;21(6):530.

[17] Shih YT , Nasso SF , Zafar SY . Price transparency for whom? In search of out‐of‐pocket cost estimates to facilitate cost communication in cancer care. Pharmacoeconomics. 2018;36(3):259‐261. doi:10.1007/s40273-018-0613-x 29396743 PMC5835199

[18] Su JA , Yeh DC , Chang CC , et al. Depression and family support in breast cancer patients. Neuropsychiatr Dis Treat. 2017;13:2389‐2396. doi:10.2147/NDT.S135624 28979126 PMC5602463

[19] Himmelstein DU , Lawless RM , Thorne D , Foohey P , Woolhandler S . Medical bankruptcy: still common despite the affordable care act. Am J Public Health. 2019;109(3):431‐433. doi:10.2105/AJPH.2018.304901 30726124 PMC6366487

[20] Gilligan AM , Alberts DS , Roe DJ , Skrepnek GH . Death or debt? National estimates of financial toxicity in persons with newly‐diagnosed cancer. Am J Med. 2018;131:1187‐1199.e5. 29906429 10.1016/j.amjmed.2018.05.020

[21] Huntington SF , Svoboda J , Doshi JA . Cost‐effectiveness analysis of routine surveillance imaging of patients with diffuse large B‐cell lymphoma in first remission. J Clin Oncol. 2015;33(13):1467‐1474. 25823735 10.1200/JCO.2014.58.5729

[22] Wadhwani N , Jatoi I . Overuse of neo‐adjuvant chemotherapy for primary breast cancer. Indian J Surg Oncol. 2020;11(1):12‐14. 32205961 10.1007/s13193-019-01002-8 PMC7064655

[23] Collins SR , Robertson R , Garber T , Doty MM . Gaps in health insurance: why so many Americans experience breaks in coverage and how the Affordable Care Act will help: findings from the Commonwealth Fund Health Insurance Tracking Survey of U.S. Adults, 2011. Issue Brief (Commonw Fund). 2012;9:1‐22. PMID: 22582451 22582451

[24] Carrera PM , Kantarjian HM , Blinder VS . The financial burden and distress of patients with cancer: understanding and stepping‐up action on the financial toxicity of cancer treatment. CA Cancer J Clin. 2018;68(2):153‐165. doi:10.3322/caac.21443 29338071 PMC6652174

[25] Yett DE , Der W , Ernst RL , Hay JW . Physician pricing and health insurance reimbursement. Health Care Financ Rev. 1983;5(2):69‐80. 10310530 PMC4191326

[26] Fitch MI , Sharp L , Hanly P , Longo CJ . Experiencing financial toxicity associated with cancer in publicly funded healthcare systems: a systematic review of qualitative studies. J Cancer Surviv. 2022;16:314‐328. 33723742 10.1007/s11764-021-01025-7

[27] Cartmell KB , Bonilha HS , Simpson KN , Ford ME , Bryant DC , Alberg AJ . Patient barriers to cancer clinical trial participation and navigator activities to assist. Adv Cancer Res. 2020;146:139‐166. 32241387 10.1016/bs.acr.2020.01.008 PMC8623462

[28] Klamerus JF , Bruinooge SS , Ye X , et al. The impact of insurance on access to cancer clinical trials at a comprehensive cancer center. Clin Cancer Res. 2010;16(24):5997‐6003. 21169253 10.1158/1078-0432.CCR-10-1451 PMC3715082

[29] Shearer GE . Confusing inequitable Medicare prescription drug benefit. J Gen Intern Med. 2007;22:286‐288. doi:10.1007/s11606-006-0080-5 17357002 PMC1824764

[30] Davis C , Darby K , Likes W , Bell J . Social workers as patient navigators for breast cancer survivors: what do African‐American medically underserved women think of this idea? Soc Work Health Care. 2009;48(6):561‐578. 19860292 10.1080/00981380902765212

[31] Dean LT , Moss SL , Rollinson SI , Frasso Jaramillo L , Paxton RJ , Owczarzak JT . Patient recommendations for reducing long‐lasting economic burden after breast cancer. Cancer. 2019;125:1929‐1940. 30839106 10.1002/cncr.32012 PMC6508994

[32] Montazeri A , Jarvandi S , Haghighat S , et al. Anxiety and depression in breast cancer patients before and after participation in a cancer support group. Patient Educ Couns. 2001;45(3):195‐198. 11722855 10.1016/s0738-3991(01)00121-5

